# Factors Associated With Voluntary Refusal of Emergency Medical System Transport for Emergency Care in Detroit During the Early Phase of the COVID-19 Pandemic

**DOI:** 10.1001/jamanetworkopen.2021.20728

**Published:** 2021-08-20

**Authors:** Nicholas E. Harrison, Robert R. Ehrman, Andrea Curtin, Damon Gorelick, Alex B. Hill, Erin Brennan, Robert Dunne

**Affiliations:** 1 Department of Emergency Medicine, Wayne State University School of Medicine, Detroit, Michigan; 2 Department of Emergency Medicine, Indiana University School of Medicine, Indianapolis; 3 Detroit Emergency Medical Control Authority, Detroit, Michigan; 4 Department of Urban Studies and Planning, Wayne State University College of Liberal Arts and Sciences, Detroit, Michigan

## Abstract

**Importance:**

Emergency department (ED) and emergency medical services (EMS) volumes decreased during the COVID-19 pandemic, but the amount attributable to voluntary refusal vs effects of the pandemic and public health restrictions is unknown.

**Objective:**

To examine the factors associated with EMS refusal in relation to COVID-19 cases, public health interventions, EMS responses, and prehospital deaths.

**Design, Setting, and Participants:**

A retrospective cohort study was conducted in Detroit, Michigan, from March 1 to June 30, 2020. Emergency medical services responses geocoded to Census tracts were analyzed by individuals’ age, sex, date, and community resilience using the Centers for Disease Control and Prevention Social Vulnerability Index. Response counts were adjusted with Poisson regression, and odds of refusals and deaths were adjusted by logistic regression.

**Exposures:**

A COVID-19 outbreak characterized by a peak in local COVID-19 incidence and the strictest stay-at-home orders to date, followed by a nadir in incidence and broadly lifted restrictions.

**Main Outcomes and Measures:**

Multivariable-adjusted difference in 2020 vs 2019 responses by incidence rate and refusals or deaths by odds. The Social Vulnerability Index was used to capture community social determinants of health as a risk factor for death or refusal. The index contains 4 domain subscores; possible overall score is 0 to 15, with higher scores indicating greater vulnerability.

**Results:**

A total of 80 487 EMS responses with intended ED transport, 2059 prehospital deaths, and 16 064 refusals (62 636 completed EMS to ED transports) from 334 Census tracts were noted during the study period. Of the cohort analyzed, 38 621 were women (48%); mean (SD) age was 49.0 (21.4) years, and mean (SD) Social Vulnerability Index score was 9.6 (1.6). Tracts with the highest per-population EMS transport refusal rates were characterized by higher unemployment, minority race/ethnicity, single-parent households, poverty, disability, lack of vehicle access, and overall Social Vulnerability Index score (9.6 vs 9.0, *P* = .002). At peak COVID-19 incidence and maximal stay-at-home orders, there were higher total responses (adjusted incident rate ratio [aIRR], 1.07; 1.03-1.12), odds of deaths (adjusted odds ratio [aOR], 1.60; 95% CI, 1.20-2.12), and refusals (aOR, 2.33; 95% CI, 2.09-2.60) but fewer completed ED transports (aIRR, 0.82; 95% CI, 0.78-0.86). With public health restrictions lifted and the nadir of COVID-19 cases, responses (aIRR, 1.01; 0.97-1.05) and deaths (aOR, 1.07; 95% CI, 0.81-1.41) returned to 2019 baselines, but differences in refusals (aOR, 1.27; 95% CI, 1.14-1.41) and completed transports (aIRR, 0.95; 95% CI, 0.90-0.99) remained. Multivariable-adjusted 2020 refusal was associated with female sex (aOR, 2.71; 95% CI, 2.43-3.03 in 2020 at the peak; aOR 1.47; 95% CI, 1.32-1.64 at the nadir).

**Conclusions and Relevance:**

In this cohort study, EMS transport refusals increased with the COVID-19 outbreak’s peak and remained elevated despite receding public health restrictions, COVID-19 incidence, total EMS responses, and prehospital deaths. Voluntary refusal was associated with decreased EMS transports to EDs, disproportionately so among women and vulnerable communities.

## Key Points

**Question:**

Were decreases in emergency medical services (EMS) and emergency department volumes associated with voluntary avoidance of emergency care during a COVID-19 outbreak in Detroit?

**Findings:**

In this cohort study of 80 487 EMS responses with intended ED transport, voluntary refusal of care was associated with lower EMS to emergency department transports during a COVID-19 outbreak from March 1 to June 30, 2020, independent of age, COVID-19 incidence, public health restrictions, and prehospital deaths. The probability of prehospital death returned to baseline when COVID-19 incidence and public health restrictions receded, but the rate of voluntary refusals remained elevated (25% in 2020 vs 15% in 2019), particularly for women and socially vulnerable communities.

**Meaning:**

The findings of this study suggest that the decreasing volume of EMS to emergency department transports was primarily associated with voluntary refusal.

## Teaser

This cohort study evaluates the use of emergency medical system transport to emergency departments during the COVID-19 pandemic.

## Introduction

Since the March 2020 declaration of a national emergency for the COVID-19 pandemic in the US, multiple reports have described reduced emergency health care use compared with previous years.^1,2,3,4,5^ Emergency department (ED) visits decreased 42% nationwide by May 2020 and remained decreased by more than 20% in the fall of 2020,^1,2,6^ despite increased visits for mental health and substance abuse.^7^ Intensive care units in New York City saw decreasing volumes of patients with stroke, heart failure, and myocardial infarction^8^ in the spring of 2020, and excess community deaths attributable to the pandemic simultaneously increased to more than 5000 in just 2 months.^9^ Emergency department visits for myocardial infarctions and strokes^1,2^ have also decreased by more than 20%. Emergency medical services (EMS) transports to EDs for emergent indications have decreased,^5,10^ and prehospital deaths have increased, including out-of-hospital cardiac arrest resuscitations terminated before ED transport and home deaths without resuscitation.^5,11,12,13^ Decreased emergency care use has been hypothesized to be due to voluntary care avoidance, increased prehospital deaths, or both. The degrees to which care avoidance vs prehospital death contribute to pandemic-era EMS volume is obscured by multiple potentially confounding factors.

First, public health measures to avoid overloading emergency care systems during the COVID-19 pandemic could decrease emergency care use in ways unrelated to COVID-19. Working from home could account for fewer EMS-transported injuries (eg, motor vehicle crashes)^5^ during COVID-19, and other communicable diseases (eg, influenza) are expected to be reduced by COVID-19 social distancing. Explicit messaging regarding the potential need to ration care, including in the media, may also encourage hospital avoidance.^14^ Second, it is difficult to draw inferences relating national emergency care use with timing of local COVID-19 surges and public health restrictions. These factors have been heterogeneous throughout different parts of the US, with varied responses before, during, and after local pandemic surges. It is therefore unclear whether decreases in EMS volume are static or instead wax and wane locally in response to community pandemic burden during an outbreak. Third, trends in higher prehospital deaths have not been studied directly alongside voluntary refusal of care. Because both events decrease EMS volume mutually exclusively, attributing relative contributions to declining emergency care use is difficult.

Few studies exist in which the choice to seek emergency care during the pandemic has been assessed directly. In one 2020 survey,^15^ 40.9% of 5412 persons reported “delayed or avoided medical care” at least once owing to the pandemic. Only 12% of respondents avoided emergency or urgent care vs 31.5% for health maintenance; however, with a response rate of 54.7%. Care avoidance is also not new,^16^ so understanding pandemic-specific care avoidance requires an adjustment for the baseline avoidance behaviors before 2020.

We studied direct refusal of EMS transport during a COVID-19 outbreak in Detroit, Michigan. The peak and nadir of COVID-19 cases during the study directly coincided with maximal and minimal public health restrictions in Detroit. Our study had 2 objectives. First, we aimed to directly quantify refusals in a large EMS system as a proportion of emergency care avoidance after accounting for public health restrictions, changes in prehospital death, preexisting rates of refusal, and other factors not directly unique to 2020 or involving a clear voluntary choice. Second, we sought to characterize temporal, geographic, and demographic factors associated with EMS refusals in 2020, adjusting for the baseline propensity of those same groups to refuse EMS in 2019.

## Methods

The Detroit East Medical Control Authority serves the City of Detroit, the Cities of Highland Park and Hamtramck (geographically within Detroit’s borders), and the affluent eastern suburbs of Grosse Pointe. Data for each EMS response in Detroit East Medical Control Authority’s 12 agencies are tracked across a catchment area of 334 US Census tracts. In this cohort study, we included 2019 and 2020 EMS responses from March 1 to June 30, excluding responses for interhospital transfers. Refusal, prehospital death, or completed hospital transport is mandatorily recorded in the database by the EMS crew for each response based on standardized definitions. Refusals are responses in which a patient voluntarily refuses transport against medical advice. Prehospital deaths are patient responses with death declared before transport, with or without resuscitation. EMS to hospital transports include total responses minus prehospital deaths and refusals. Age, sex, date, and address were geocoded to Census tracts using US Census Bureau TIGER/LINE public data sets. The project was determined exempt by the Wayne State University Institutional Review Board, including a waiver of informed consent because data are required to be collected and analyzed routinely for the quality assessment and improvement responsibilities of the Detroit East Medical Control Authority. This study followed the Strengthening the Reporting of Observational Studies in Epidemiology (STROBE) reporting guideline for cohort studies.^17^

Social determinants of health have been implicated in care avoidance before COVID-19.^16^ To capture community social determinants of health as a risk factor for death or refusal, we paired each response’s address with the Centers for Disease Control and Prevention’s Social Vulnerability Index (SVI) 2018 Census Tract data set.^18^ The Centers for Disease Control and Prevention’s SVI consists of 15 risk factors for a community to experience disproportionately negative public health outcomes when confronted with a natural disaster or disease outbreak, such as a recent study of vaccination disparities in the COVID-19 pandemic.^18^ Four domain subscores and the overall SVI score (0-15, with higher scores indicating greater vulnerability) are based on these variables (eMethods in the Supplement). All 15 SVI variables, including race/ethnicity, are determined by the Centers for Disease Control and Prevention from Census data and chosen as known social determinants of health.

The March 1 to June 30, 2020, period was chosen because it allowed modeling of COVID-19 incidence and public health restrictions through 2020 temporal trends. A late-March peak and mid-June nadir represent the overall high and low points of COVID-19 incidence in Detroit at the time of writing. Public health measures at this peak and nadir also represented extremes of restrictions vs reopening of the city. Given closely associated timing for COVID-19 incidence and restrictions during the study period, we modeled the combined outcomes of both as a function of date. We compared 2020 vs 2019 daily responses, completed transports, prehospital deaths, and refusals to evaluate their associations with age, sex, community resilience (by SVI), and the natural history of this single severe COVID-19 outbreak, including a comparison at the outbreak’s peak and nadir. A date vs date comparison was deemed suitable because no signs of seasonality or trend were noted on 2019 time series analysis (eMethods in the Supplement).

### Statistical Analysis

**Figure 1.**
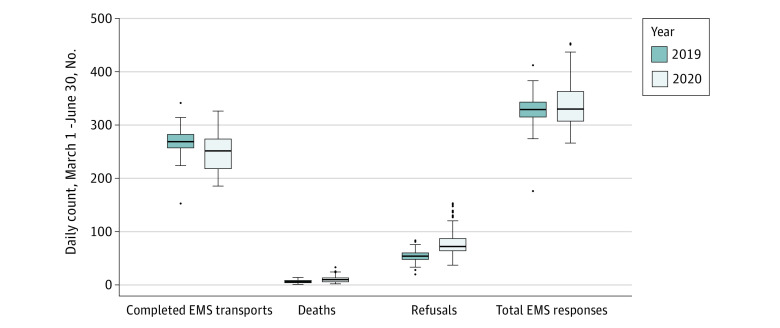
Emergency Medical Services (EMS) Use Total EMS responses, completed EMS to hospital transports, prehospital deaths, and refusals of emergently indicated EMS transport in 2019 vs 2020, reported as daily count for March 1 to June 30, 2020. EMS to hospital transports equal total EMS responses minus prehospital deaths and refusals. Solid line indicates median; box, interquartile range (IQR); whiskers, 1.5 times the IQR; and individual dots, outliers.

Descriptive statistics are reported for high-refusal Census tracts in 2020 vs low-refusal tracts using the Wilcoxon rank sum test. We defined high-refusal tracts as those with refusals per population at or above the median and low refusal as below the median. Mean differences in 2020 vs 2019 with 95% CIs for daily counts of responses, deaths, refusals, and transports were calculated for the entire study period (Figure 1) and for smaller date segments in which a significant change in temporal trend was detected for each time series in 2020 (eMethods, eFigures 2-4 in the Supplement).

Differences between 2019 and 2020 were adjusted for age, sex, total SVI score, and date. Multivariable models were fit and estimates were obtained using the rms package in R, version 3.6.1 (R Foundation).^19^ Total responses and hospital transports were modeled with Poisson regression to obtain estimated daily counts per Census tract and adjusted incident rate ratios (aIRRs) for covariate effects on 2020 vs 2019 daily counts per tract. Refusals and deaths were modeled with logistic regression as the mutually exclusive odds of either event occurring for a single response. An attempt was made to include EMS agency in the model as a random effect, but model convergence and estimation were not possible under these conditions; thus, each multivariable model was fit under fixed effects. Covariate effects are reported as adjusted odds ratios (aORs) and estimates as daily probabilities. Subgroup analysis was considered significant if the 95% CI of a subgroup’s 2020 vs 2019 aOR crossed the aOR estimate of effect when estimating the multivariable model at the mean value of continuous variables and stratified by each level of categorical variable (eg, sex and peak vs nadir); the eMethods in the Supplement provides further details on regression methods. All statistical analyses were conducted with the R programming language (R Foundation for Statistical Computing). A 2-sided *P* < .05 was considered statistically significant.

## Results

From March 1 through June 30, 2020, there were 40 984 EMS responses in 2020 and 39 503 in 2019, with 1299 vs 760 prehospital deaths and 9601 vs 6463 voluntary refusals of emergency transport (62 636 completed EMS to ED transports). Of patients included in the analysis, 38 621 (48%) were women, mean (SD) age was 49.0 (21.4) years, and mean (SD) SVI score was 9.6 (1.6). Daily responses (mean difference, 10.1; 95% CI, 1.4-18.8; *P* < .001), deaths (mean difference, 4.4; 95% CI, 3.4-5.5; *P* < .001), and refusals (mean difference, 25.2, 95% CI, 20.4-30.1; *P* < .001) were all higher in 2020 vs 2019 (Figure 1). Completed hospital transports decreased (mean difference, −19.5; 95% CI, −26.6 to −12.5; *P* < .001). Mean total SVI score was 9.6 (range, 2.7-12.9) (eFigure 1A in the Supplement), with 2020 patients being older (mean [SD], 49.8 [21.2] vs 47.7 [21.6] years; *P* < .001) and less often female (47% vs 50%, *P* < .001). Summary demographics are presented for Census tracts (Table 1) and patients (Table 2). High-refusal tracts compared with low-refusal tracts had higher Census tract percentiles for people living below the poverty line (88.2% vs 77.0%), unemployed (88.4% vs 78.3%), older than 65 years (41.1% vs 33.8%), with a disability (80.7% vs 58.6%), in a single-parent household (80.9% vs 71.7%), of a minority race/ethnicity (94.3% vs 85.8%), in a multiunit household (62.8% vs 47.2%), and without a vehicle (91.2% vs 78.2%). High-refusal tracts had lower percentiles of persons younger than 18 years (57.2% vs 67.7%) or with limited English proficiency (19.6% vs 41.9%). Social Vulnerability Index level was higher (9.8 vs 9.0; *P* = .002) in high-refusal tracts vs low-refusal tracts (eFigure 1B and 1C in the Supplement).

**Table 1.** Unadjusted Comparison Between Census Tracts in Detroit in 2020 With EMS Refusals per Population Greater or Less Than the Median^a^

SVI variable	US Census percentile or SVI score		*P* value
	High-refusal tracts (n = 167)	Low-refusal tracts (n = 167)	
Below federal poverty line	88.2	77.0	<.001
Unemployment	88.4	78.3	<.001
Per capita income	13.6	25.8	.03
No high school diploma	83.3	73.5	.36
Age, y			
≥65	41.1	33.8	.005
≤17	57.2	67.7	.001
Living with a disability	80.7	58.6	<.001
Single-parent household	80.9	71.7	.001
Minority race or ethnicity	94.3	85.8	<.001
Limited English proficiency	19.6	41.9	<.001
Mobile homes	16.0	18.7	.43
Multiunit households	62.8	47.2	<.001
Crowded households	51.2	56.8	.06
No vehicle available to household	91.2	78.2	<.001
Group quarters living	43.1	37.4	.17
No health insurance	9.2	9.7	.71
SVI theme			
1: Socioeconomic status (0-4)	3.5	3.0	<.001
2: Household composition (0-4)	2.6	2.3	<.001
3: Race/ethnicity/language (0-2)	1.1	1.3	.02
4: Housing/transportation (0-5)	2.6	2.4	.009
Overall SVI (0-15)	9.8	9.0	.002

Abbreviations: EMS, emergency medical services; SVI, Social Vulnerability Index.

^a^ Population greater than the median indicates high refusal; population less than the median indicates low refusal.

**Table 2.** Unadjusted Comparison of 2020 vs 2019 EMS Responses, Deaths, and Refusals

Variable	Total EMS responses			Refusals			Deaths		
	All (N = 80 487)	2020 (n = 40 984)	2019 (n = 39 503)	All (N = 16 064)	2020 (n = 9601)	2019 (n = 6463)	All (N = 2059)	2020 (n = 1299)	2019 (n = 760)
Female, No. (%)	38 621 (48)	19 318 (47)	19 553 (49)	8532 (53)	5097 (53)	3435 (53)	778 (38)	504 (39)	274 (36)
Age, mean (SD), y	49 (21.4)	49.8 (21.2)	47.7 (21.6)	47 (22.6)	47.6 (22.3)	45 (22.9)	62 (19.2)	62.7 (19.1)	60.2 (19.2)
Total SVI score, mean (SD)^a^	9.6 (1.6)	9.7 (1.5)	9.6 (1.6)	9.6 (1.5)	9.7 (1.4)	9.5 (1.6)	9.7 (1.6)	9.7 (1.6)	9.6 (1.6)

Abbreviations: EMS, emergency medical services; SVI, Social Vulnerability Index.

^a^ The SVI contains 4 domain subscores; possible overall score is 0 to 15, with higher scores indicating greater vulnerability.

The daily count mean differences in 2020 vs 2019 during the maximal time segments of responses (92.8; 95% CI, 80.0-105.6) (eFigure 2 in the Supplement, segment 2), deaths (12.8; 95% CI, 10.8-14.9) (eFigure 3 in the Supplement, segment 2), and refusals (76.8; 95% CI, 68.8-84.8) (eFigure 4 in the Supplement, segment 3) were significantly more pronounced than overall mean differences for March 1 to June 30 (Figure 1). All 3 factors began concurrent decreases with downward-trending COVID-19 incidence after the March 29 peak. Total responses decreased the most sharply (eFigure 2 in the Supplement, segment 3), briefly decreasing below the 2019 baseline in mid-April (−27; 95% CI, −41.0 to −27.9) (eFigure 2 in the Supplement, segment 4) before returning to baseline from mid-May onward when reopening began (−4.6; 95% CI, −14 to 4.8) (eFigure 2 in the Supplement, segment 5). Deaths decreased more slowly but followed total responses in reaching the 2019 baseline at the nadir of the outbreak in June (0.0; 95% CI, −1.7 to 1.7) (eFigure 3 in the Supplement, segment 4). By contrast, a brief decrease in refusals following the COVID-19 peak leveled in mid-April and remained higher than in 2019 (17.5; 95% CI, 14.1-20.8) (eFigure 4 in the Supplement, segment 4) through June 30 despite the decreasing COVID-19 incidence and progressively relaxed public health restrictions through the ensuing 2.5 months.

**Figure 2.**
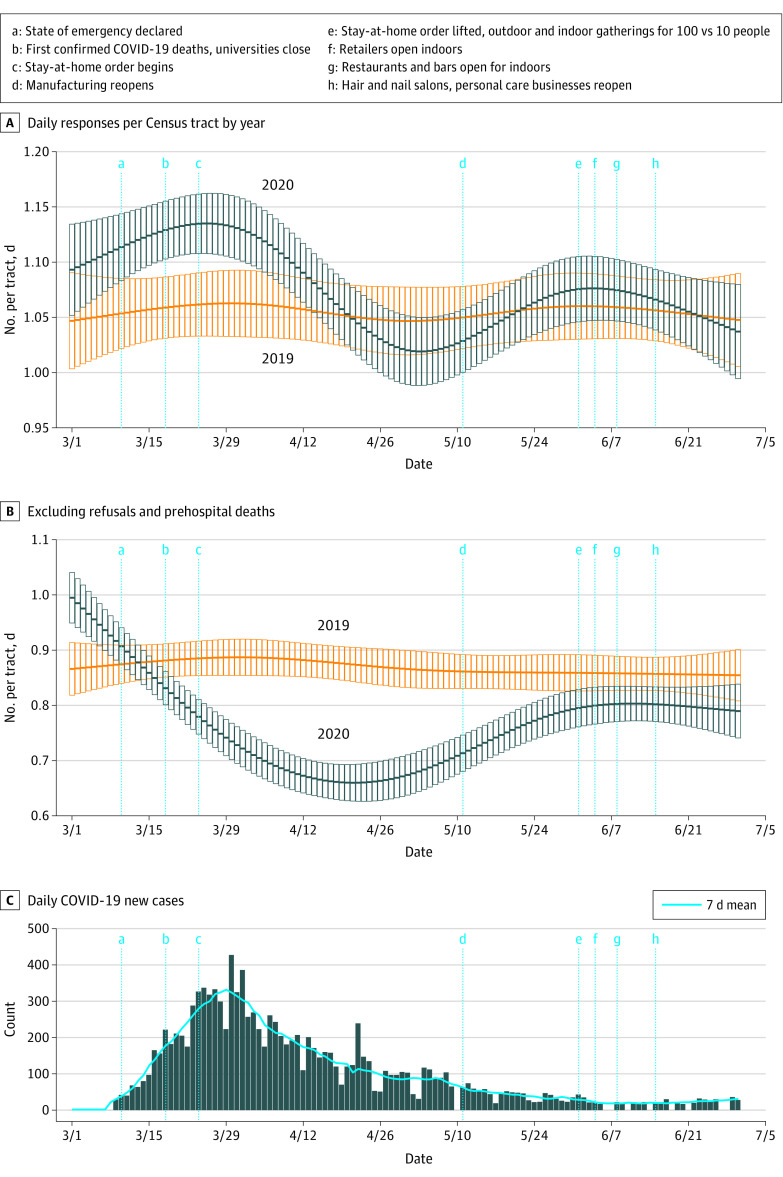
Emergency Medical Services Responses Over Time A, Multivariable-adjusted mean daily count per Census tract for emergency medical services responses. B, Daily counts excluding emergency medical services refusals and prehospital deaths. C, New daily cases of COVID-19 (bars) and 7-day moving average (solid blue line) are shown for the same Census tracts across the same dates. Counts with error bars (95% CIs) are shown for each day in the study period. Major milestones in public health restrictions being initiated (a-c) or relaxed (d-h) are indicated by vertical blue lines across all 3 graphs.

**Figure 3.**
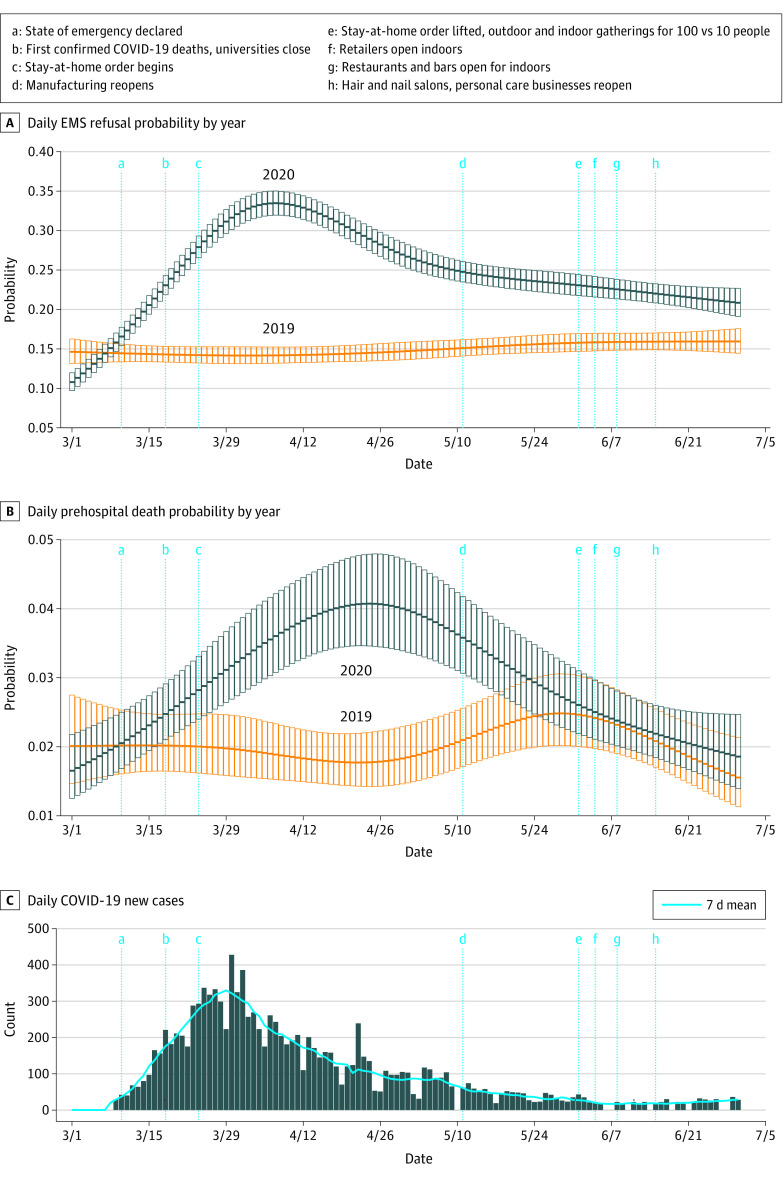
Emergency Medical Services (EMS) Probability of Refusal or Transport Multivariable-adjusted mean daily probability for an EMS response ending in voluntary refusal of emergency transport (A) or prehospital death (B) from March to June 2019 and 2020. The probability of refusal was for all EMS responses in which EMS transport was completed or voluntarily refused (ie, prehospital deaths are excluded in panel A). Prehospital death probability is for all EMS responses resulting in completed transport or prehospital death (ie, excludes refusals). Probability with error bars (95% CI) are shown for each day in the study period. C, COVID-19 new daily cases (bars) and 7-day moving average (solid blue line) are shown for the same Census tracts across the same dates. The trend of 2020 probability of prehospital death follows the trend in COVID-19 incidence closely, with peaks and decreases delayed by a few weeks; 2020 deaths approximate the 2019 baseline as COVID-19 incidence and public health restrictions reach a nadir. Conversely, refusal probability shows a more modest decrease after the COVID-19 peak and little change in response to reopening events (d-h).

Figure 2 shows daily EMS responses (total and transported) per Census tract for 2020 vs 2019 after adjusting for date, age, sex, and SVI. Figure 3 shows the daily probability of a response ending in death or refusal, adjusted for the same factors.

At peak COVID-19 incidence and public health restrictions (March 29), the aIRR for total responses in 2020 vs 2019 was 1.07 (95% CI, 1.03-1.12), equating to 23.9 more responses per day in 2020. After excluding refusals and deaths, 48.4 fewer completed hospital transports were occurring per day (aIRR,  0.82; 95% CI, 0.78-0.86). Deaths (aOR, 1.60; 95% CI, 1.20-2.12) and refusals (aOR, 2.33; 95% CI, 2.09-2.60) were both more likely in 2020 at this peak in COVID-19 and public health restrictions.

At the nadir of COVID-19 incidence and with broad reopening (June 20), there was no significant difference in total responses (aIRR, 1.01; 95% CI, 0.97-1.05; 2.4 more responses per day in 2020). The odds of deaths in 2020 was similar to 2019 at the nadir (aOR, 1.07; 95% CI, 0.81-1.40). By contrast, daily hospital transports remained significantly decreased (aIRR, 0.95; 95% CI, 0.90-0.99; 18.6 fewer transports per day in 2020), and refusals remained more likely (aOR, 1.27; 95% CI, 1.14-1.40) at the nadir in 2020.

The overall March 1 to June 30 probability of refusing EMS transport was 25% in 2020 compared with just 15% in 2019. This differential was as high as 33% vs 14% during the late March peak and was still high (22% vs 15%) at the nadir when restrictions were lifted, COVID-19 incidence was minimal, and deaths and total responses returned to baseline.

On subgroup analysis (eFigure 5 in the Supplement), sex and SVI were associated with refusal probability in 2020. Women had a higher odds of refusal compared with the mean for 2020 (aOR, 2.71; 95% CI, 2.43-3.03 at the peak; aOR; 1.47; 95% CI, 1.32-1.64 at the nadir). Responses to less socially vulnerable Census tracts were less likely to end in refusal at the nadir, with an association noted at an SVI score of approximately 6 or less (eFigure 5 in the Supplement). The 2020 refusal odds did not vary significantly by age, and there were no significant subgroups by age, sex, or SVI score for the aOR of prehospital death (eFigure 6 in the Supplement).

## Discussion

In this study, we examined the direct evidence of care avoidance through refusals of EMS to hospital emergency transports after adjusting for COVID-19 community incidence, social distancing restrictions, a concomitant increase in out-of-hospital deaths, and demographic factors.

Although earlier reports have suggested avoidance of emergency care by observing decreased ED^2,3^ or EMS volumes,^5^ to our knowledge, this is the first report to measure emergency care avoidance directly through voluntary refusal of medically recommended emergency care. This distinction is important because the decrease in both EMS and ED volumes during the pandemic could be influenced by numerous plausible factors other than a voluntary choice to avoid emergency care. Lerner et al^5^ noted a decrease in EMS responses across the US for injuries, which could be due to stay-at-home orders that limited motor vehicle crashes and other trauma. By evaluating cases in which a voluntary choice to refuse care was made, we were able to directly quantify care avoidance behavior associated with the pandemic. We observed a substantial difference: the chance of refusing EMS transport in 2020 was more than 1 in 4 compared with 1 in 7 before the pandemic, which is a greater than 10% higher absolute rate of refusal. This increase equates to 1 further refusal in 2020 for every 10 EMS responses.

Numerous reports have documented an increase of prehospital deaths in the COVID-19 era,^5,11,13^ including in Detroit,^12^ and our study adds to that body of evidence. In addition, our results suggest that both prehospital deaths and voluntary refusals contribute to declining EMS to ED transports but that refusals had less association with daily changes in the pandemic case load and restrictions. This finding is best evidenced by the fact that, at the nadir of our COVID-19 outbreak when deaths and total EMS responses had returned to their 2019 baselines, there remained a substantially lower rate of transport associated with significantly higher refusals.

Our study also lends context to the complex relationship between emergency care volumes and a local case surge. Although total EMS responses were overall similar in 2019 and 2020, short-term variations fluctuated substantially (Figure 2). At the peak COVID-19 incidence, responses were much greater in 2020, but this increase was offset by reduced responses once COVID-19 cases began to decrease and public health restrictions were still in full effect. In contrast, mean daily refusals increased at the peak of cases and restrictions and remained increased after cases decreased and restrictions were lifted. Thus, refusals appear to be at least partly influenced by factors beyond just case burden and public health measures.

Women and individuals from more socially vulnerable Census tracts were more likely to refuse, including at the nadir in COVID-19 cases and restrictions (Figure 3). The Census tracts accounting for the most refusals in 2020 had higher unemployment, more single-parent households, and more persons of a racial or ethnic minority (Table 1). These findings raise a few possibilities.

First, social determinants of health are associated with the choice to refuse emergency care, suggested by the fact that low overall social vulnerability was a protective factor at the nadir (eFigure 5 in the Supplement). With COVID-19 cases and restrictions at a minimum, refusals remained high in socially vulnerable Census tracts and overall but were close to 2019 levels in the least vulnerable tracts (eFigure 5 in the Supplement). Marginalized communities before the pandemic were already less likely to have access to health care facilities^20^ and more likely to rely on emergency care safety nets.^21^ Numerous social considerations exist regarding health care use.^22^ Our results suggest a need for public health agencies to focus outreach regarding emergency care avoidance in the most marginalized communities, including identification of barriers to seeking emergency care and identifying the unmet needs of communities caused by the pandemic.

Second, the apparently disproportionate effect on women and in Census tracts with more single parents could reflect the equally disproportionate negative consequences of the pandemic on working women. Women were also more likely to refuse EMS transport in 2019, but after adjusting for this baseline propensity, the association between female sex and refusal probability was even greater in 2020. This difference suggests that the pandemic exacerbated a preexisting social condition with implications for refusal among women. A lack of expansive childcare services in the US has been long-standing but was exacerbated for many families during the pandemic. This increased difficulty may have disproportionately affected women, as those who work full time in the US spend an average of 50% more time on childcare than full-time working men.^23^ Women have also disproportionately left the labor force during the pandemic, with 80% of those leaving in September 2020 being female and the effects on women in racial and ethnic minority groups being even more pronounced.^24^ It is possible that worsened unmet childcare needs during the pandemic led women to refuse emergency care more often. A higher rate of single-parent households in high-refusal tracts would seem to support this theory. Further investigation of the attitudes of parents, particularly mothers and single parents, is needed to examine whether such an effect is present.

Third, higher rates of refusals in Census tracts with more residents belonging to racial or ethnic minority groups have several possible explanations. Detroit has a large African American population and, given documented disparities in COVID-19 infections and outcomes for African American individuals, it is possible that this group disproportionately avoided the hospital as a result.^25,26,27^ Alternatively, many Census tracts in Detroit with a high proportion of African American individuals have high social vulnerability from determinants other than race.

### Limitations

Our study has several additional limitations. Given collinearity between individual components of the SVI, our models were not powered to detect subtle differences between individual correlated SVI subcomponents (eg, race and income), a limitation of the study. Further research should aim at determining which specific factors of social vulnerability are most independently associated with EMS refusal. A before-after design risks the possibility that the baseline year was unusual compared with a typical year. Also, our choice of March through June may have missed important changes before Michigan’s first confirmed COVID-19 case. However, most studies looking at EMS and ED volumes during COVID-19 have used similar methods,^2,3,5,11,13^ and there is no reason to suspect that 2019 was substantially unusual. Moreover, to decrease the chance that unforeseen factors specific to 2019 affected EMS use, we chose a period of study determined to be stationary for both trend and seasonality in 2019 by formal statistical methods of time series analysis (eMethods and eAppendix in the Supplement). For an unforeseen factor to significantly skew 2019 as a baseline compared with other years, the factor would have to affect the entire March through June time series monotonically and without trend. Although this confounding is possible, it is unlikely. A lack of data on medical comorbidities is another limitation because people with severe comorbidities could be expected to avoid care more often. Because there is a dearth of research directly assessing the factors associated with EMS refusal, we can only speculate that more detail on medical comorbidities and other factors, such as the mix of presenting problems, would have further enriched our models. For instance, the national increases in overdose ED visits represent generally high-refusal encounters, and mental health visits^7^ represent generally low-refusal encounters. The nature of our data set precluded such an analysis, and these data should be evaluated in future investigations. Our data are also specific to Detroit and may not generalize to other cities. However, our catchment area contains substantial demographic and clinical diversity, yielding a sample size of over 80 000 patients. Among the 12 EMS agencies, differences in refusal or death could have occurred, and we were unable to account for this factor in the present analysis owing to statistical complexity in the model and the data set. In addition, we can only state that total social vulnerability at the community level was a risk factor for EMS refusal but not more specific individual factors. Although the unadjusted analysis can give some hints to important specific determinants, collinearity between the individual components of the SVI prevented meaningful regression analysis based on the component factors. A prospective study directly capturing these individual variables on patients refusing transport would therefore add value.

## Conclusions

In this cohort study, we found overall that care avoidance in the form of voluntary EMS to hospital transport refusal was substantially higher during the COVID-19 pandemic, with 1 additional refusal in 2020 for every 10 EMS responses, after adjusting for baseline refusal propensity and demographic confounders. High COVID-19 incidence and public health restrictions were associated with increasing refusals at the peak of an outbreak, but refusal odds remained increased once pandemic conditions improved. This finding differed from the response of total EMS responses and prehospital deaths, whose trends more closely matched the ebb and flow of COVID-19 cases and public health restrictions. Sex and social vulnerability were significantly associated with higher 2020 refusals vs 2019 but not prehospital deaths. Our results suggest that EMS refusals have a complex temporal association with the direct effects of the pandemic and that care avoidance behavior in the COVID-19 era may be rooted in social factors affecting the most vulnerable populations.

## Notes

Supplement. **eFigure 1.** Social Vulnerability Index (SVI) for the 334 Census Tracts Served by the Detroit East Medical Control Authority (DEMCA) **eFigure 2.** Time Series Analysis of EMS Responses **eFigure 3.** Time Series Analysis of Prehospital Deaths **eFigure 4.** Time Series Analysis of EMS Refusal **eFigure 5.** Multivariable Model of Refusal Odds in 2020 vs 2019 **eFigure 6.** Multivariable Model of Death Odds in 2020 vs 2019 **eMethods.** Detailed Methods **eAppendix.** Detailed Results
