# Supplementary material for: Factors Associated With Voluntary Refusal of Emergency Medical System Transport for Emergency Care in Detroit During the Early Phase of the COVID-19 Pandemic
Source: JAMA Netw Open. 2021 Aug 20;4(8):e2120728. doi: 10.1001/jamanetworkopen.2021.20728 (PMC8379656; doi:10.1001/jamanetworkopen.2021.20728)
Supplement: Supplement. — eFigure 1. Social Vulnerability Index (SVI) for the 334 Census Tracts Served by the Detroit East Medical Control Authority (DEMCA) eFigure 2. Time Series Analysis of EMS Responses eFigure 3. Time Series Analysis of Prehospital Deaths eFigure 4. Time Series Analysis of EMS Refusal eFigure 5. Multivariable Model of Refusal Odds in 2020 vs 2019 eFigure 6. Multivariable Model of Death Odds in 2020 vs 2019 eMethods. Detailed Methods eAppendix. Detailed Results [file jamanetwopen-e2120728-s001.pdf]

Supplementary Online Content

Harrison NE, Ehrman RR, Curtin A, et al. Factors associated with voluntary refusal of emergency medical system transport for emergency care in Detroit during the early phase of the COVID-19 pandemic. *JAMA Netw Open*. 2021;4(8):e2120728. doi:10.1001/jamanetworkopen.2021.20728

eFigure 1. Social Vulnerability Index (SVI) for the 334 Census Tracts Served by the Detroit East Medical Control Authority (DEMCA)

eFigure 2. Time Series Analysis of EMS Responses

eFigure 3. Time Series Analysis of Prehospital Deaths

eFigure 4. Time Series Analysis of EMS Refusal

eFigure 5. Multivariable Model of Refusal Odds in 2020 vs 2019

eFigure 6. Multivariable Model of Death Odds in 2020 vs 2019

eMethods. Detailed Methods

eAppendix. Detailed Results

This supplementary material has been provided by the authors to give readers additional information about their work.

© 2021 Harrison NE et al. *JAMA Network Open.*

eFigure 1: Social Vulnerability Index (SVI) for the 334 census tracts served by the Detroit East Medical Control Authority (DEMCA)

DEMCA is the medical control authority for the City of Detroit and the municipalities of Hamtramck, Highland Park, and Grosse Pointe. The SVI is a 0 to 15 index of a census-tract community's vulnerability to adverse public health outcomes in the event of a pandemic or natural disaster. The DEMCA catchment area contains a stark disparity between pockets of affluent low-vulnerability tracts (e.g. Grosse Point in the Northeast, Downtown/Midtown Detroit in the South along the Detroit River, the gated communities of Palmer Woods in the North, etc.) amongst a background of highly vulnerable communities in the ≥80th national percentile of SVI.

1A. All 334 Census Tracts By SVI

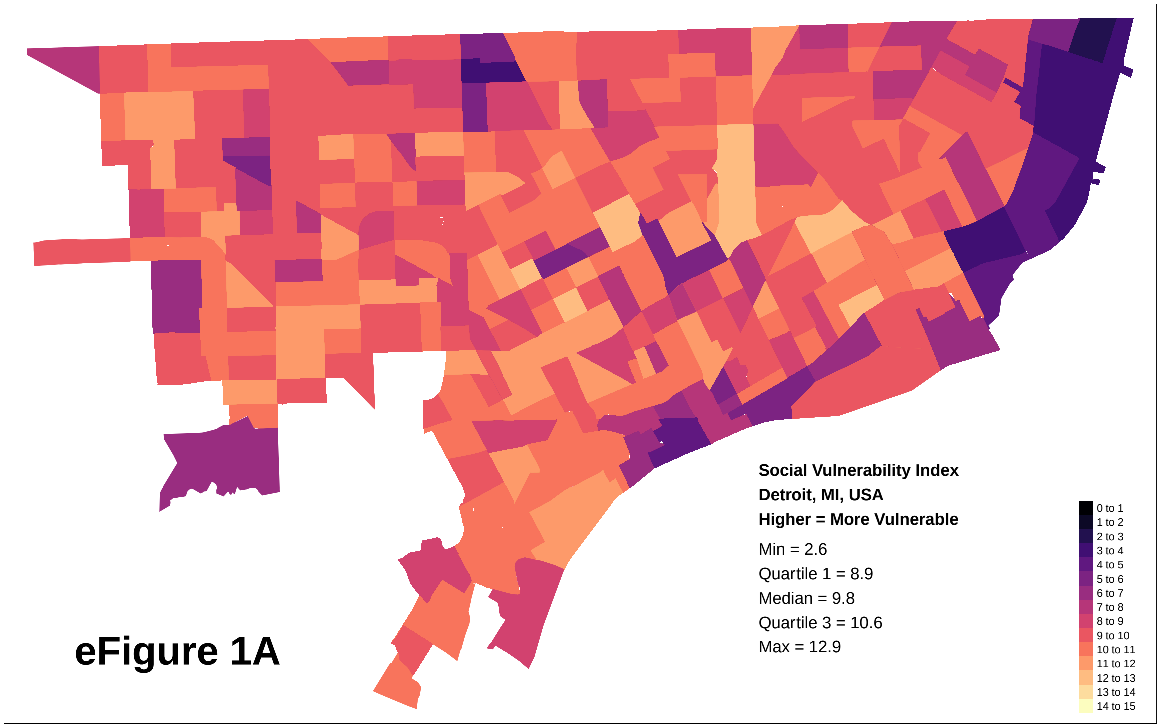

© 2021 Harrison NE et al. *JAMA Network Open.*

1B. High Refusal Census Tracts

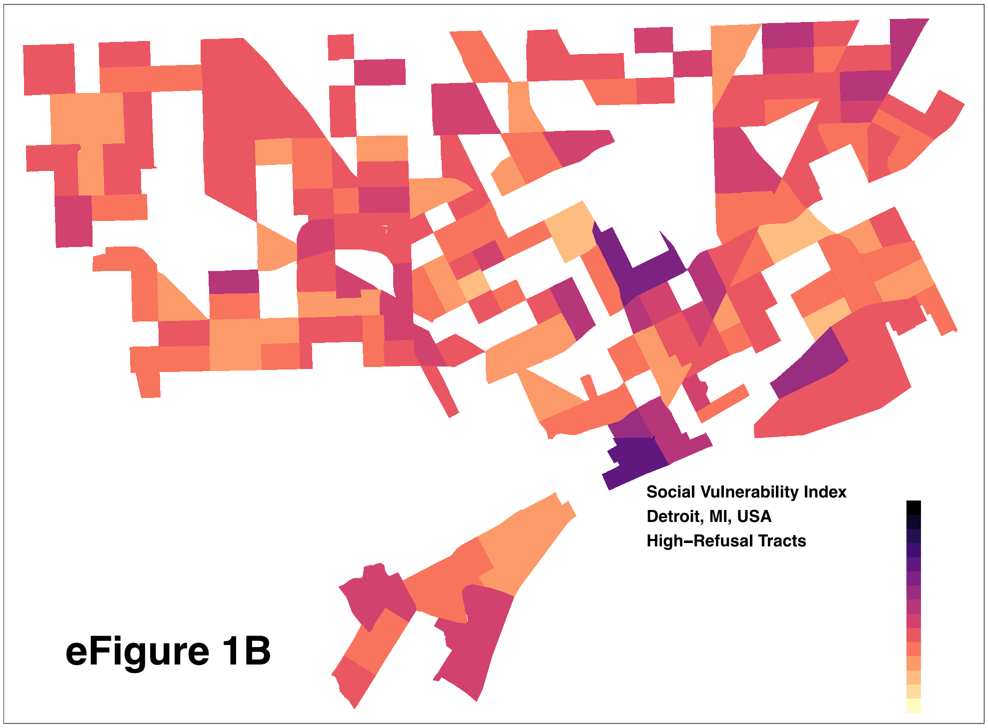

1C. Low Refusal Census Tracts

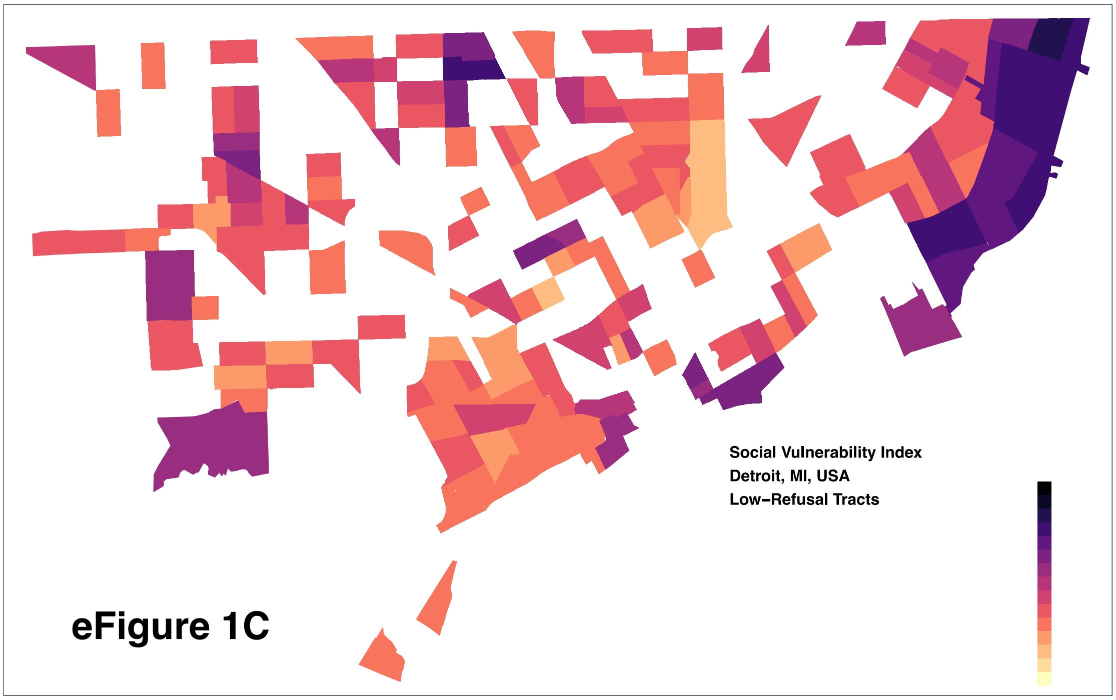

© 2021 Harrison NE et al. *JAMA Network Open.*

eFigure 2: Time series analysis of EMS responses

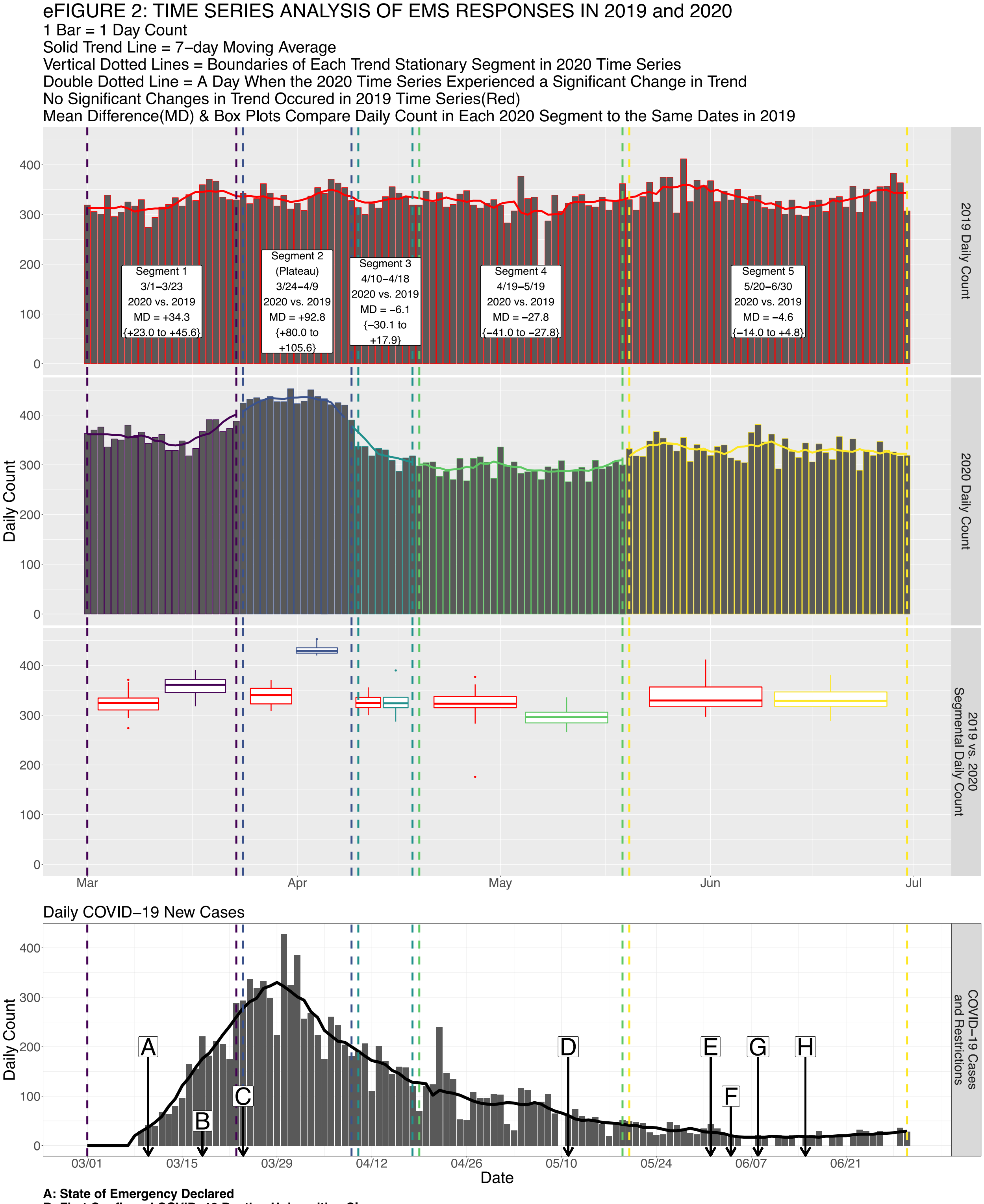

A: State of Emergency Declared
B: First Confirmed COVID−19 Deaths, Universities Close
C: Stay−at−home Order Begins
D: Manufacturing Reopens
E: Stay−at−home Lifted, Outdoor and Indoor Gatherings for 100 vs. 10 people
F: Retailers Open Indoors
G: Restaurants & Bars Open For Indoors
H: Hair & Nail Salons, Personal Care Businesses Reopen

© 2021 Harrison NE et al. *JAMA Network Open.*

Total daily EMS responses in 2019 (red) showed no signs of seasonality or trend (KPSS test $p>0.05$, no changepoints detected) over the March - June study period (bars = daily count, line = 7-day moving average). By contrast, four changepoints in the 2020 time series mean daily responses were identified, corresponding to five significantly different (KPSS $p<0.05$) segments. COVID-19 incidence and selected public health milestones are shown for comparison (bottom). The segment-by-segment comparison shows that 2020 responses substantially exceeded 2019 early in the COVID-19 surge, but this was offset by 2019 exceeding 2020 in the middle (green) of the time series. Comparison of daily counts for the total time period (Figure 1) were mildly higher for total responses, but the segmental analysis here shows that this overall 2020 vs. 2019 comparison masks subtle significant trends within the time series.

© 2021 Harrison NE et al. *JAMA Network Open.*

eFIGURE 3: TIME SERIES ANALYSIS OF PRE−HOSPITAL DEATHS IN 2019 and 2020

1 Bar = 1 Day Count
Solid Trend Line = 7−day Moving Average
Vertical Dotted Lines = Boundaries of Each Trend Stationary Segment in 2020 Time Series
Double Dotted Line = A Day When the 2020 Time Series Experienced a Significant Change in Trend
No Significant Changes in Trend Occured in the 2019 Time Series (Red)
Mean Difference(MD) & Box Plots Compare Daily Count in Each 2020 Segment to the Same Dates in 2019

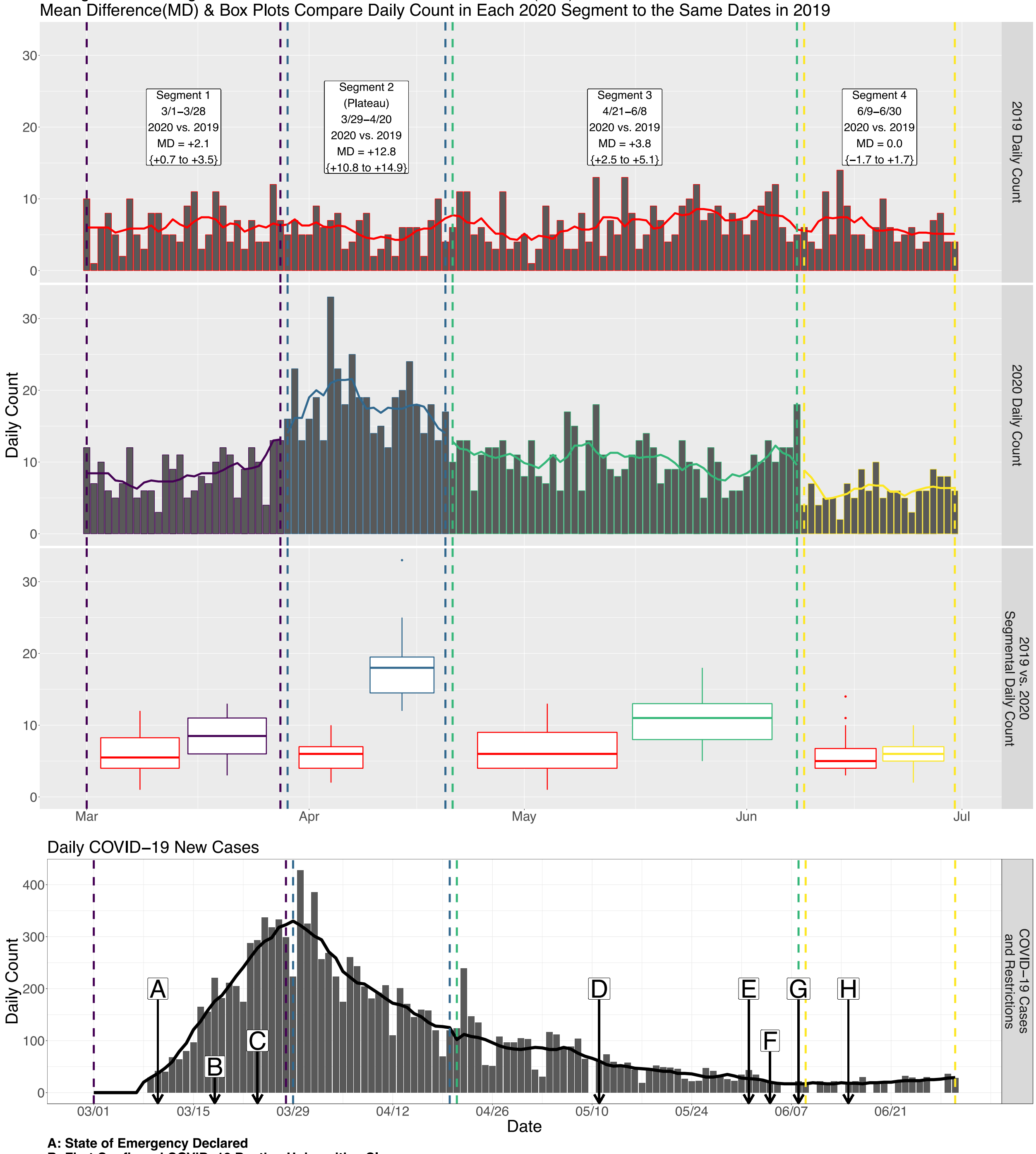

A: State of Emergency Declared
B: First Confirmed COVID−19 Deaths, Universities Close
C: Stay−at−home Order Begins
D: Manufacturing Reopens
E: Stay−at−home Lifted, Outdoor and Indoor Gatherings for 100 vs. 10 people
F: Retailers Open Indoors
G: Restaurants & Bars Open For Indoors
H: Hair & Nail Salons, Personal Care Businesses Reopen

© 2021 Harrison NE et al. *JAMA Network Open.*

Daily pre-hospital deaths in 2019 (red) showed no signs of seasonality or trend (KPSS test p>0.05, no changepoints detected) over the March - June study period (bars = daily count, line = 7-day moving average). By contrast, three changepoints in the 2020 time series mean daily deaths were identified, corresponding to four segments with significantly different trends(KPSS p<0.05). COVID-19 incidence and selected public health milestones are shown for comparison (bottom). The segment-by-segment comparison shows that 2020 deaths substantially exceeded 2019 beginning 2-3 weeks after a corresponding rise in COVID-19 incidence. Deaths similarly showed a 2–3 week delayed decline corresponding to COVID-19 incidence declining, and by the nadir of COVID-19 have returned to baseline. Comparison of daily counts for the total time period (Figure 1) showed more pre-hospital deaths, but the segmental analysis here shows that this overall 2020 vs. 2019 comparison masks subtle significant trends within the time series.

© 2021 Harrison NE et al. *JAMA Network Open.*

eFigure 4 - Time series analysis of EMS refusal

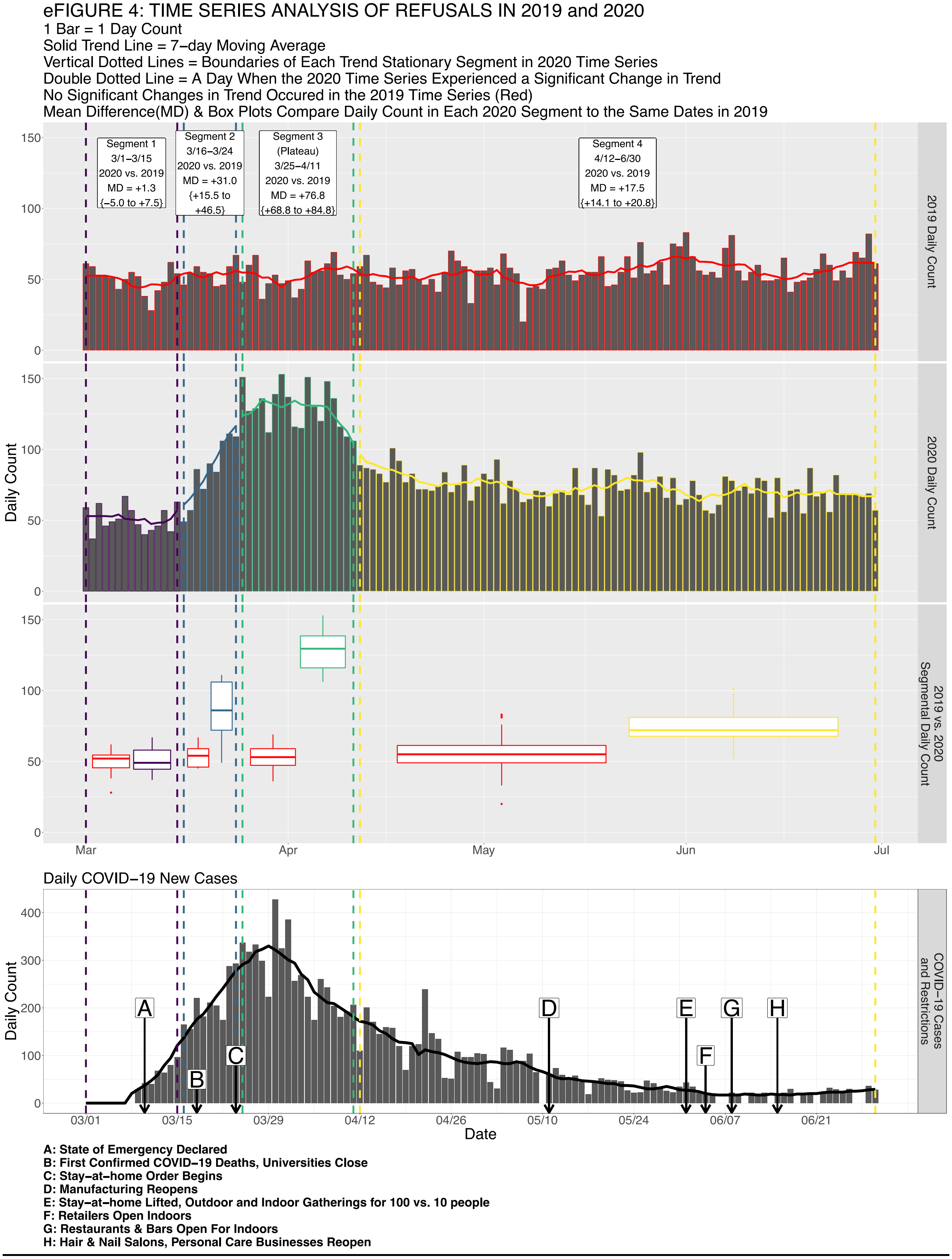

© 2021 Harrison NE et al. *JAMA Network Open.*

Daily EMS refusals in 2019 (red) showed no signs of seasonality or trend (KPSS test $p>0.05$, no changepoints detected) over the March - June study period (bars = daily count, line = 7-day moving average). By contrast, three changepoints in the 2020 time series of mean daily refusals were identified, corresponding to four segments with significantly different (KPSS $p<0.05$) trends. COVID-19 incidence and selected public health milestones are shown for comparison (bottom). The segment-by-segment comparison shows that 2020 refusals substantially exceeded 2019 rates, and show modest correlation to a peak in COVID-19 incidence. Unlike total EMS responses (eFigure 2) or pre-hospital deaths (eFigure 3), daily refusals remain elevated once there is a decline in COVID-19 incidence and lifting of public health restrictions. This finding for the unadjusted daily refusal count persists in the evaluation of multivariable-adjusted refusal probability (Figure 3 in the main manuscript).

© 2021 Harrison NE et al. *JAMA Network Open.*

eFigure 5

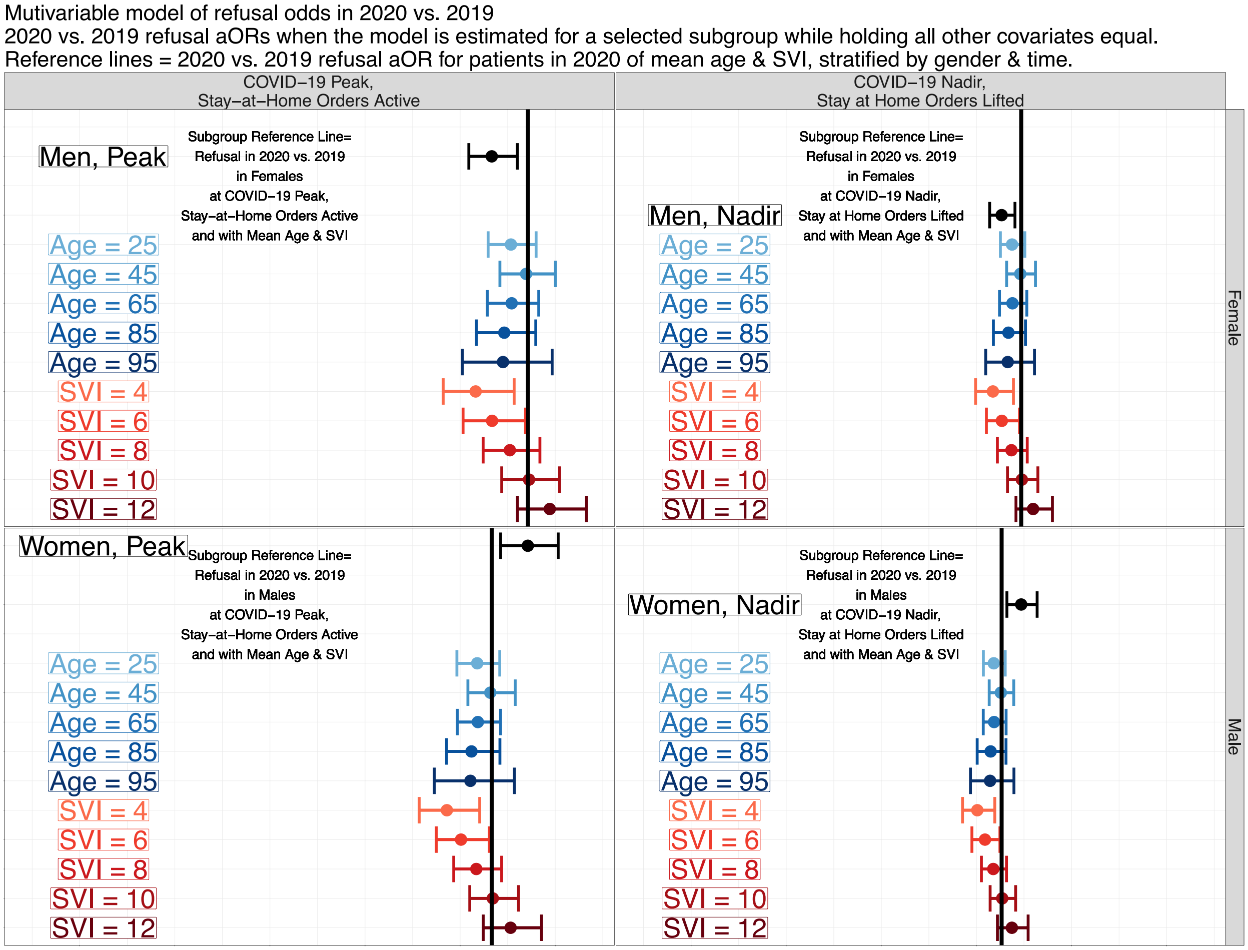

Subgroup effects are compared for the peak vs. nadir of COVID-19 incidence and public health restrictions (left vs. right), and for women vs. men (top vs. bottom). Adjusted odds ratios (points) with their 95% confidence (error bars) are presented at subgroup slices in each quadrant. The solid black vertical line is the mean effect for the reference group in the quadrant: therefore, a statistically significant subgroup effect exists when a given subgroup's error bar does not cross the black line. At both the peak and nadir, female gender was significantly associated with higher odds of refusal in 2020. Adjusting for this gender difference, no significant difference effect of age on refusal was noted compared to the overall 2020 refusal odds (for both peak and nadir). By contrast, the odds of refusing transport in 2020 vs. 2019 was less pronounced in patients with low social vulnerability index (SVI ≤ 6) compared to overall. At the nadir, 95% confidence for SVI ≤ 6 not only showed a significant subgroup difference (solid black line), estimates in these groups also crossed the point of no 2020 vs. 2019 effect (i.e. aOR = 1, line of no effect not pictured). Thus, at the nadir, patients with a low SVI in 2020 were more similar to 2019 patients, while higher refusal rate persisted for those with moderate to high SVI > 6.

© 2021 Harrison NE et al. *JAMA Network Open.*

eFigure 6

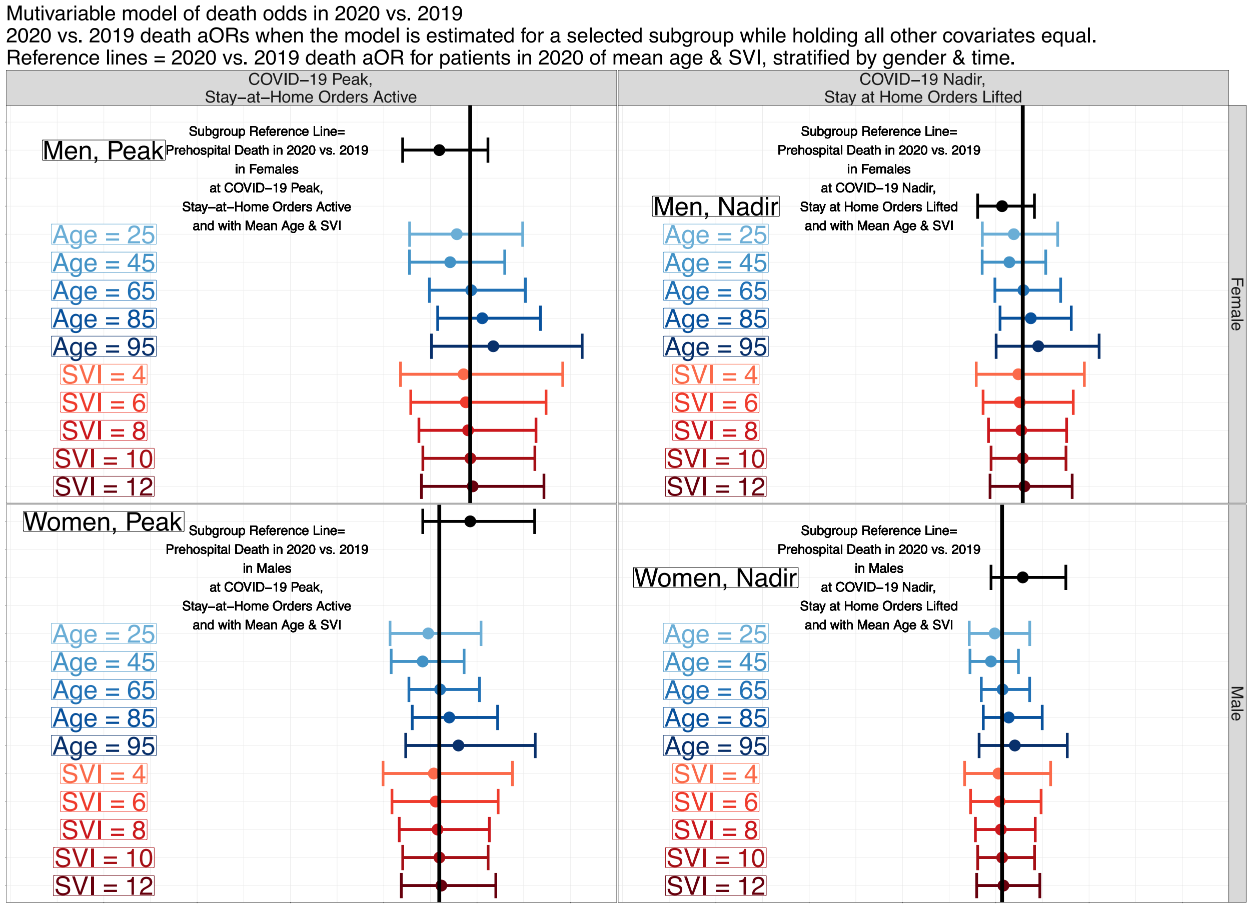

Subgroup effects for the multivariable adjusted probability of prehospital death in 2020 vs. 2019 are shown, with similar interpretation of the forest plots here as in Figure 5. In contrast to the subgroup effects by gender and social vulnerability index (SVI) on refusal probability in Figure 5, no significant subgroup effects on pre-hospital death probability are noted with regards to age, gender, or SVI. In other words, the propensity for pre-hospital death in 2020 was overall significantly higher than 2019, but the 2020 vs. 2019 difference was not accounted for by changing demographics between those years in our analysis. Point estimates do show a trend towards higher adjusted odds of 2020 death with increasing age and female gender, so it is possible that modest differences could exist below what the analysis was powered to detect.

© 2021 Harrison NE et al. *JAMA Network Open.*

e Methods: Detailed Methods

SVI variables, domain scores, and total score description

The 15 component variable scores come from the US Census American Community Survey (ACS), 2014-2018 (5-year) estimates. Variables are reported as percentiles, and further summarized in one of four domain scores: socioeconomic status (scores 0 - 4), household composition (0 - 4), race/ethnicity/language (0 - 2), housing/transportation (0 - 5). A total SVI score (0 - 15) is then calculated from the sum of all 4 domains/15 individual variables. In all cases a lower score corresponds to lower risk (i.e. lower social vulnerability) of adverse health outcomes in a disease outbreak compared to a higher score (more social vulnerability). See more details at https://www.atsdr.cdc.gov/placeandhealth/svi/index.html.

Timeline of COVID-19 incidence and public health restrictions in Detroit

The first confirmed case of COVID-19 in Detroit occurred March 10th. Public health restrictions including a strict stay-at-home order were enacted concurrently as incidence rose. The 7-day average of daily new cases reached a peak March 29th, and the stay-at-home order and other restrictions were in full effect March 24th. At this peak, Michigan had more COVID-19 cases and deaths than any state other than New York and New Jersey, with the vast majority in Detroit. COVID-19 incidence in Detroit steadily declined thereafter, accompanied by a progressive relaxation of public health restrictions. This began May 11th with manufacturing, followed by retail business appointments and elective medical procedures in late May, the stay-at-home order and prohibitions on indoor and outdoor gatherings on June 1, indoor retail without appointment June 4th, indoor bar and restaurant service June 8th, and personal care such as hair and nail salons on June 15th. Concurrently, the steady decline in COVID-19 incidence reached a nadir June 6th - June 17th.

Assessment of the 2019 time series as a suitable baseline

March - June 2019 was deemed suitable for a date vs. date comparison because this period included no effects of seasonality or trends unique to 2019 for responses, deaths, or refusals. By contrast, 3 changepoints for trend in daily count were identified in the 2020 death and refusals time series, and 4 in the 2020 responses time series. Thus, March 1 - June 30 2019 represented a baseline for which comparisons to the same dates in 2020 would only reflect temporal trends specific to 2020 (e.g. a change in the trend of responses, deaths, or refusals in response to rising/falling COVID-19 cases and restrictions).

Additionally, given no seasonality or trend in 2019, segmental time periods of interest in 2020 for each time series (responses, deaths, or refusals) were compared to their corresponding dates in 2019 for MDs as well (eFigures 2-4). This segmental analysis was performed to initially describe the degree to which 2020 vs. 2019 differed not just overall, but for significant periods of interest in the natural history of the pandemic (e.g. the early March 2020 rise of refusals after the first COVID-19 death was announced, the plateau in refusals from mid-April to June 30th 2020, etc.).

© 2021 Harrison NE et al. *JAMA Network Open.*

Mitigating seasonality as a confounder enhances the inferential strength of a before-after analysis, since like all such studies the analyses are predicated on the assumption that outcome rate and trajectory in the baseline year is unaffected by unmeasured external forces to a significant degree (i.e. that 2019 is like any other year). To determine if there was any point in 2019 where the trajectory of responses, deaths, and/or refusals by daily count changed, each time series was analyzed with changepoint detection (changepoint package in R) and the Kwiatkowski-Phillips-Schmidt-Shin {KPSS} test. Optimal partitioning by pruned exact linear time[28] was used with the CROPS algorithm[29] to identify if, and when, a change in daily mean occurred. Penalty weights for the CROPS algorithm were obtained from plotting and examining elbow plots from each time series to avoid over or under-fitting of changepoint detection. Any changepoints identified, along with the absolute beginning (March 1) and end (June 30) of each time series, were considered to be a boundary for a stationary segment of the larger time series. Segments were checked for stationarity by the KPSS test, with a p-value of ≤0.05 used to identify any segments in which the assumption of trend-stationarity was violated. Any non-stationary segments identified by the KPSS test were examined and lengthened or shortened by 1 day until the entire time series contained only trend-stationary segments. Each segment therefore represented a period in time during which the trend of the time series mean for that year was homogenous, with each changepoint representing a change in trajectory due to the onset of seasonality or the effect of an external variable (e.g. rising/falling COVID-19 cases and public health restrictions). No changepoints or other signs of trend or seasonality occurred for responses, deaths, or refusals in 2019.

<u>*Additional Regression Modeling Details*</u>

We chose logistic modelling for refusals and deaths rather than Poisson, because the counts of both were expected to be heavily influenced by fluctuations in EMS volumes between different days and geographies. The logistic model for refusal probability was fit after excluding deaths from the dataset, since voluntary refusal and prehospital death are mutually exclusive events. Variables were assessed for their ideal restricted cubic spline or monotonic transformations, and multiply imputed for missing predictors, with the utilities of the rms package by previously described methods [18]. Less than 0.5% of observations contained missing data, including none for any outcomes, dates, or refusal vs. death designations. Regression response variables were modeled with an interaction between year 2020 vs. 2019 and all covariates; and reported as adjusted incident rate ratios (aIRR) and adjusted odds ratios (aOR) for the Poisson and logistic model covariates (respectively). aIRRs and aORs for overall 2020 vs. 2019 comparisons, as well as covariate subgroup effects on the outcome, were assessed at the COVID-19 peak and COVID-19 nadir using the summary.rms and Predict functions of the rms package [18].

© 2021 Harrison NE et al. *JAMA Network Open.*

eAppendix. Detailed Results

Time series analysis of trend-stationary segments for responses, deaths and refusals in 2020 vs. 2019

During the study period in 2019 there were no statistically significant changes in the daily rates of responses, deaths, or refusals. All three 2019 time series remained flat and without trend or evidence of seasonality for the entire March 1 - June 30 period. In 2020, by contrast, the trend in daily responses changed 4 times, while the trends in responses and refusals changed 3 times each (eFigures 2-4). Responses, deaths, and refusals all rose in 2020 in close relation to the trajectory of COVID-19 cases in March, with a rise and peak in deaths lagging slightly behind refusals and total responses. Responses and deaths regressed to their 2019 baseline by the nadir of COVID-19 incidence and public health restrictions. Refusals declined slightly from their peak as incidence and restrictions waned, but this decline halted abruptly in mid-April. After this, a persistently higher 2020 refusals compared to 2019 were noted, regardless of further declines in incidence and increasing reopening.

© 2021 Harrison NE et al. *JAMA Network Open.*
